# Characterising protective immune responses to SARS-CoV-2 in urban and rural Malawi between February 2021 and April 2022

**DOI:** 10.1038/s41598-025-22599-7

**Published:** 2025-10-29

**Authors:** Mhairi J. McCormack, Louis Banda, Stephen Kasenda, Ellen C. Hughes, Lina Leonhard, Annie Mwale, Estelle McLean, Alison Price, Amelia Crampin, David Chaima, Abena S. Amoah, Tonney S. Nyirenda, Antonia Ho, Brian J. Willett

**Affiliations:** 1 MRC-University of Glasgow Centre for Virus Research, Glasgow, UK; 2 Malawi Epidemiology and Intervention Research Unit (MEIRU), Lilongwe, Malawi; 3 Department of Livestock and One Health, Institute of Infection, Veterinary and Ecological Sciences, University of Liverpool, Liverpool, UK; 4 Public Health Institute of Malawi, Lilongwe, Malawi; 5 London School of Hygiene and Tropical Medicine, London, UK; 6 School of Health and Wellbeing, University of Glasgow, Glasgow, UK; 7 Kamuzu University of Health Sciences (KUHeS), Blantyre, Malawi; 8 Leiden University Medical Center, Leiden, Netherlands

**Keywords:** SARS-CoV-2, Seroepidemiology, Malawi, Neutralising antibody, Longitudinal, HIV, Viral infection, Epidemiology

## Abstract

SARS-CoV-2 transmission in Malawi remains unclear due to high proportions of mild/asymptomatic infections and limited diagnostics. Existing seroprevalence studies in Malawi have primarily used convenience samples and enzyme-linked immunosorbent assays (ELISAs). We assessed SARS-CoV-2 neutralisation in a longitudinal Malawian population-based cohort, assessing protective immunity post-infection and vaccination. Sera were obtained from rural (Karonga, *n* = 958) and urban (Lilongwe, *n* = 918) based participants at three-monthly intervals (February 2021-April 2022). Neutralising antibodies against SARS-CoV-2 were measured using human immunodeficiency (HIV)-based pseudotype assays in HIV-uninfected participants, and vesicular stomatitis virus-based assays in HIV-infected participants and an HIV-uninfected subset. Nucleocapsid ELISAs identified vaccinated participants also infected. SARS-CoV-2 neutralisation profiles increased in complexity over time from rising vaccination coverage and emerging variants. Neutralising antibody prevalence was higher in Lilongwe than Karonga (68.1% (CI 63.5–72.4) vs. 45.4% (CI 41.6–49.3), Survey 4). Hybrid immune and solely vaccinated participants exhibited higher titres than those solely infected. Children < 15 years had the lowest neutralising antibody titres among infected (not vaccinated) participants. People living with HIV had lower neutralising responses than those HIV-uninfected, particularly post vaccination. We therefore recommend surveillance of children and people living with HIV as low neutralisation responses increase reinfection risk. COVID-19 vaccination should be prioritised for HIV-infected individuals.

**Supplementary Information:**

The online version contains supplementary material available at 10.1038/s41598-025-22599-7.

## Introduction

SARS-CoV-2, the virus responsible for the COVID-19 pandemic, has posed significant public health challenges worldwide. While reported cases and associated deaths in sub-Saharan Africa, including Malawi, have been comparatively lower, it remains unclear whether this reflects a genuinely milder impact or limited detection and reporting^1^, highlighting the need to study the virus in diverse regions. The first COVID-19 cases in Malawi were reported in April 2020 in the capital city, Lilongwe^2^. By April 2022, approximately 86,000 confirmed cases were recorded in the country^3^, however this is likely a gross underestimate due to limited routine SARS-CoV-2 diagnostic testing^4^ and a high proportion of asymptomatic or pauci-symptomatic infections^5^. Malawi experienced four SARS-CoV-2 waves in the two years post emergence: the B.1 ancestral virus (June-August 2021); Beta (December 2020-April 2021); Delta (June-September 2021); and Omicron BA.1/BA.2 (December 2021-January 2022)^6^. Notably, Malawi was one of the few African countries that did not implement a nationwide lockdown^7,8^.

Malawi’s national COVID-19 vaccination campaign began in March 2021^9^. Vaccination of key workers, the elderly, and those with comorbidities was prioritised, later expanding to all adults and adolescents^9^. Initially, only the ChAdOx1 nCoV-19 (Oxford AstraZeneca) vaccine (AZ) and Ad26.COV2.S (Johnson & Johnson) vaccine (J&J) were available in Malawi. By April 2022, 9.1% of all adults/adolescents had received AZ-one dose, while 8.4% were fully vaccinated (AZ-two doses/J&J-one dose)^3^. Booster doses had been administered to 4,388 individuals^3^.

Serosurveillance studies aid in understanding SARS-CoV-2 exposure in areas with limited testing. Existing SARS-CoV-2 serosurveillance studies in Malawi have aimed to characterise the seroprevalence in this under vaccinated country, finding very low levels of seropositivity (9.3%^10^, 18.5%^11^ at the end of 2020. These rates increased greatly by July 2021, particularly in urban settings (Blantyre – 81.7%, Mzuzu 71.0%) with neutralisation against the ancestral B.1 virus and Beta variant being detected^11^. Later, the Omicron variant emerged resulting in further increases to seroprevalence, in combination with increasing vaccination coverage^12^. However, these studies existing were limited by either their cross-sectional designs^12^, or reliance on convenience samples (i.e., health care workers^10^ and blood donors^11^.

The COVSERO study addressed some of these limitations by examining SARS-CoV-2 IgG responses in population-based rural and urban cohorts in Malawi (February 2021-April 2022), using enzyme-linked immunosorbent assays (ELISAs)^6^. While ELISAs detect antibodies that bind to viral proteins, they do not measure virus neutralisation. Neutralisation assays measure neutralising antibodies (nAbs) that prevent the virus from infecting cells, indicative of protective immunity^13^. Additionally, with limited sequencing data from Malawi, these assays help infer variant exposure. Here, we expand on the COVSERO study, investigating the cohort’s neutralisation responses using pseudotyped virus neutralisation assays (PVNAs). With this, we characterised the protective immunity afforded by infection and vaccination, and examined key variables that influence nAb strength.

## Results

### Participant characteristics

Data from 1,876 participants were included: Karonga (rural), *n* = 958 (51.1%, CI 48.8–53.3); Lilongwe (urban), *n* = 918 (48.9%, CI 46.7–51.2). The median age was higher in Lilongwe (26.7 years, IQR 14.8–41.1) than Karonga (21.2 years, IQR 11.8–39.8) (*p* = 0.00028, Wilcoxon) (Table 1). In terms of biological sex, Lilongwe had more females (61.8%, CI 58.6–64.9) compared with Karonga (50%, CI 46.8–53.2) (*p* < 0.0001, chi-sq). Lilongwe had higher rates of diabetes (*p* = 0.016, chi-sq), hypertension (*p* = 0.00079, chi-sq), and people living with HIV (*p* = 0.0090, chi-sq) (Table 1). Asthma (*p* = 0.0010, chi-sq) and heart disease (*p* = 0.0070, Fisher) were more prevalent in Karonga (Table 1). The median follow up time between study surveys was 106 days (minimum – 44 days, maximum – 222 days). COVID-19 vaccination coverage was low at Survey 1 (Karonga, 3.4% (CI 2.3–4.9); Lilongwe 9.2% (CI 7.3–11.5)) with participants having only received one dose of the AZ vaccine (Supplementary Fig. 1). By Survey 4, this increased to 23.6.% (CI 20.6–27.0) in Karonga and 30.8% (CI 26.8–35.3) in Lilongwe, with participants having received AZ-one or two doses and J&J-one dose, with one dose of J&J being considered fully vaccinated with a primary course. Vaccination coverage was consistently higher in Lilongwe (Survey 1, *p* < 0.0001; Survey 2, *p* = 0.021; Survey 3, *p* = 0.0003; Survey 4, *p* = 0.0085; chi-sq) (Supplementary Fig. 1).

**Table 1:** Participant characteristics at baseline. For continuous variables, the p-value was determined using the Wilcoxon rank-sum test. For categorical variables where both cell counts >=5, a chi-squared test was used, while a Fisher’s exact test was used if at least one cell count was <5.

	Karonga (rural) (*n* = 958)	Lilongwe (urban) (*n* = 918)	*P*-value
Median age (IQR), years	21.2 (11.8–39.8)	26.7 (14.8–41.1)	0.00028
Age category, years - n (%)	.	.	< 0.0001
< 15	326 (34.0)	231 (25.2)	.
15–39	394 (41.1)	440 (47.9)	.
40–59	178 (18.6)	173 (18.8)	.
≥ 60	60 (6.3)	74 (8.1)	.
Sex assigned at birth - n (%)	.	.	< 0.0001
Female	479 (50.0)	567 (61.8)	.
Male	479 (50.0)	351 (38.2)	.
Comorbidities - n (%)	.	.	.
Asthma	77 (8.0)	37 (4.0)	0.0010
Cancer	1 (0.1)	3 (0.3)	0.59
Chronic kidney disease	5 (0.5)	0 (0.0)	0.081
Chronic lung disease (not asthma)	0 (0.0)	2 (0.2)	0.084
Diabetes	5 (0.5)	18 (2.0)	0.016
Heart disease	17 (1.8)	4 (0.4)	0.0070
Hypertension	51 (5.3)	87 (9.5)	0.00079
Liver disease	3 (0.3)	0 (0.0)	0.026
Stroke	5 (0.5)	1 (0.1)	0.024
Tuberculosis (past or current)	7 (0.7)	16 (1.7)	0.013
HIV	36 (3.8)	60 (6.5)	0.0090

*IQR* - interquartile range, *HIV - * human immunodeficiency virus.

### Increasing complexity of SARS-CoV-2 neutralisation profiles over time

Neutralisation responses to SARS-CoV-2 evolved greatly over the course of the study, indicating changes in protective immunity in the cohort. To accurately assess the nAb responses in the cohort, the following analyses were restricted to HIV-uninfected participants (*n* = 1,780), as an alternative assay was required for measurement of neutralisation in HIV-infected participants due to interference with the HIV base of the pseudotypes. Overall, nAb prevalence increased over time, being consistently higher in Lilongwe than Karonga (*p* < 0.0001–all surveys, chi-sq) (Supplementary Fig. 2). At Survey 1, 6.7% (CI 5.2–9.9) were nAb positive in Karonga, compared with 14.5% (CI 12.0-17.3) in Lilongwe. By Survey 4, this increased to 45.4% (CI 41.6–49.3) in Karonga and 68.1% (CI 63.5–72.4) in Lilongwe (Supplementary Fig. 2).

**Fig. 1:**
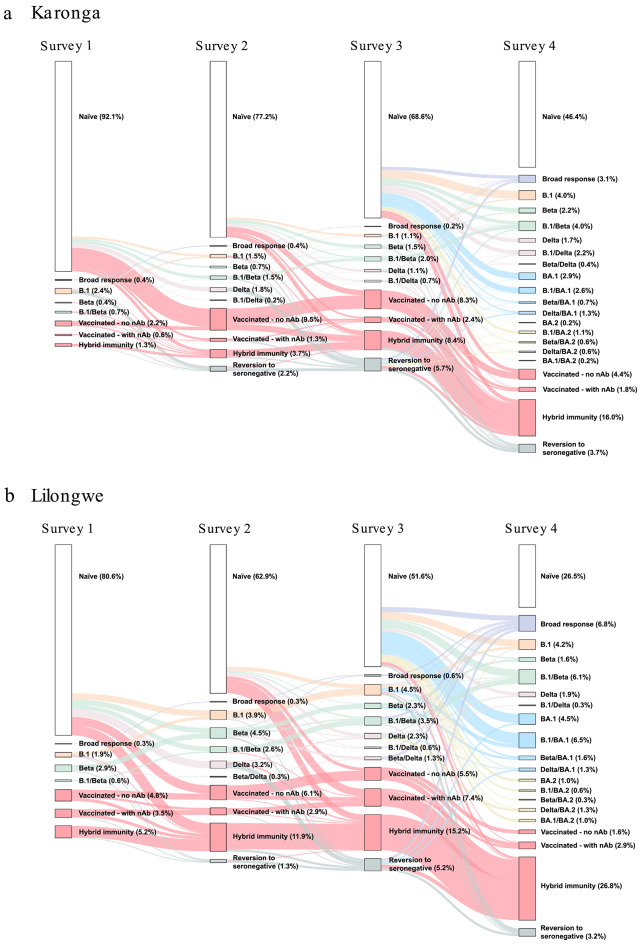
Distribution of SARS-CoV-2 immunological profiles in urban and rural Malawi. Sankey plot displaying the distribution of SARS-CoV-2 immunological protective immunity profiles at each study survey and the transition across categories between the surveys. Sankey plots for (**A**) the rural site - Karonga, and (**B**) the urban site - Lilongwe. Plots include responses from HIV-uninfected participants who had a complete sample series (Karonga, *n* = 545; Lilongwe, *n* = 310, total, *n* = 855). Each rectangular node represents the proportion of the population with a specific SARS-CoV-2 exposure, determined by the dominant titre observed with HIV(SARS-CoV-2) PVNA. White colour represents those who were naïve to SARS-CoV-2 (no nAb response, not vaccinated); purple represents those who had a broad SARS-CoV-2 response, with approximately equal titres to 3 or more variants; orange represents those who were ancestral virus (B.1)-dominant; green represents those who were Beta-dominant (including individuals with dominant responses to Beta as well as a variant that occurred before the Beta wave, i.e. pre Beta); pink represents those who were Delta-dominant (including Delta plus pre-Delta responses); blue represents those who were Omicron BA.1-dominant (including BA.1 plus pre-BA.1 responses); yellow represents those who were Omicron BA.2-dominant (including BA.2 plus pre-BA.2 responses). Those who had been vaccinated are shown in red, this includes those vaccinated with and without neutralising responses, as well as those with both infected and vaccinated - determined by Nucleocapsid ELISA). Grey represents those who reverted to a seronegative status i.e., those who at a previous study survey tested positive for nAbs but subsequently tested negative.

In both study sites, when looking at participants sampled across the entire study period, the SARS-CoV-2 neutralisation profiles became increasingly complex as vaccination coverage increased, and variants emerged (Fig. 1). At Survey 1, infected participants had ancestral B.1 virus- and Beta-dominant responses. By Survey 2, Delta-dominant responses were detected - Karonga 2.0% (CI 1.1–36), Lilongwe 3.5% (CI 2.0-6.2) (including individuals with dominant responses to Delta and a variant that occurred before the Delta wave). By Survey 4, Omicron BA.1/BA.2 had emerged. In Karonga, 7.5% (CI 5.6–10.0) were Omicron BA.1-dominant and 2.7% (CI 1.5–4.3) were Omicron BA.2-dominant, while in Lilongwe, 13.9% (CI 10.1–18.2) were Omicron BA.1-dominant and 4.2% (CI 2.5-7.0) were Omicron BA.2-dominant (including individuals with dominant responses to BA.1/BA.2 and a variant that occurred before the Omicron wave).

Hybrid immunity was determined by looking at those vaccinated who were positive when tested with the Nucleocapsid (N) ELISA and therefore had also been naturally infected. Rates of hybrid immunity increased over time, reaching 16.0% (CI 13.1–19.3) in Karonga and 26.8% (CI 22.2–32.0) in Lilongwe at Survey 4 (*p* = 0.0004; chi-sq) (Fig. 1). Rates of hybrid immunity were consistently higher in Lilongwe (Survey 1, *p* = 0.0022; Survey 2, *p* < 0.0001; Survey 3, *p* = 0.0035; chi-sq), (Fig. 1). The proportion of vaccinated individuals who had no detectable nAbs peaked at Survey 2 (Karonga, 9.5% (CI 7.4–12.3); Lilongwe 6.1% (CI 4.0-9.4)) then decreased by Survey 4 (Karonga, 4.4% (CI 3.0-6.5); Lilongwe, 1.6% (CI 0.1–3.7)), as many developed hybrid immunity. Among vaccinated participants in each survey who were HIV-uninfected (not just those with complete sample series displayed in Fig. 1), the proportion who were N ELISA positive generally increased, with a substantial rise in infections at Survey 4 in particular (Survey 1: 36% (34/94); Survey 2: 41% (80/195); Survey 3: 33% (79/239); Survey 4: 73% (194/267)).

Rates of reversion to seronegative status (when an individual who previously tested nAb positive subsequently tested negative, i.e. seroreverted) peaked at Survey 3 (Karonga, 5.7% (CI 4.0–8.0); Lilongwe, 5.2% (CI 3.2–8.2)) (Fig. 1). Seroreversion was highest among Delta-dominant individuals (Fig. 1). Of those who seroreverted at Survey 3, 29% (CI 16.1–46.6) in Karonga and 38% (CI 18.5–61.4) in Lilongwe had previous Delta-dominant responses at Survey 2.

### Trajectory of nAb responses over the study period

**Fig. 2:**
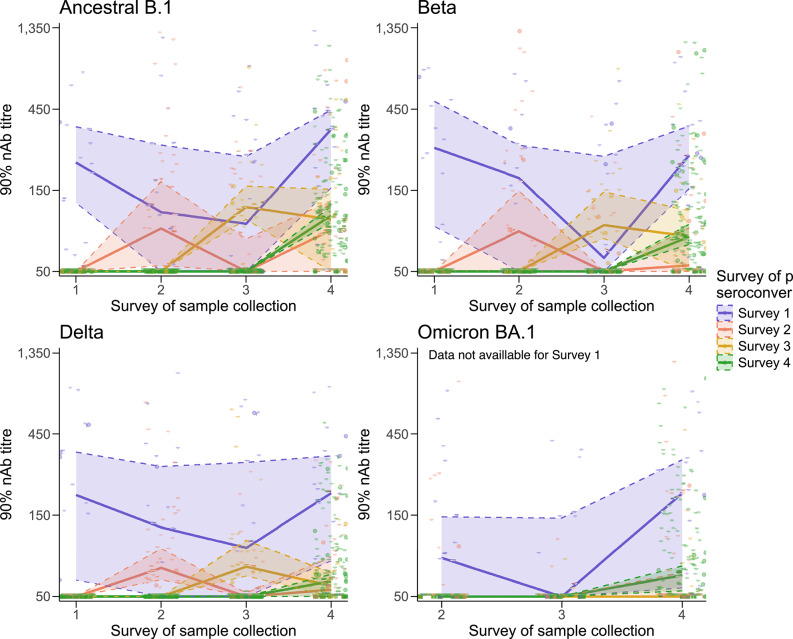
Longitudinal analysis of neutralising antibody responses. Neutralising antibodies (nAb) in participants who were naturally infected with SARS-CoV-2 decreased after their first infection and then increased following subsequent infections. Participants included in this analysis are those HIV-uninfected and COVID-19 unvaccinated, who were positive for SARS-CoV-2 nAbs at least once over the study surveys, but had a complete sample series (*n* = 215). The x-axis displays the study survey of sample collection. The y-axis displays the 90% nAb titres measured with the HIV(SARS-CoV-2) PVNA. Participants were grouped by the survey of participant seroconversion (i.e. when the first nAb positive was detected per individual): Survey 1 – *n* = 18 (blue); Survey 2 – *n* = 39 (red); Survey 3 – *n* = 24 (yellow); Survey 4 – *n* = 134 (green). Solid line represents the median titre at each survey per seroconversion participant group. Shaded area (coloured by survey converted at, as before) displays the 95% confidence interval (CI) of the median. Omicron BA.1 responses were only measured at Surveys 2–4. Alpha and Omicron BA.2 were excluded as they were only tested against at Survey 1 and Survey 4, respectively.

Many individuals showed similar responses to multiple variants, complicating dominant variant assignment. To explore if this stemmed from multiple infections, we examined nAb titres over time in infected participants who contributed a complete sample series, grouped by the survey at which individuals seroconverted. Post initial exposure, median titres declined by the following survey then increased at Survey 4, likely due to Omicron infections (Fig. 2). Individuals who were seropositive at Survey 1 (ancestral B.1/Beta-dominant) generally exhibited the strongest and most sustained nAb responses, with the magnitude of boosting being particularly high after Omicron reinfection (Survey 4) (Fig. 2).

We then looked at the individual-level changes in nAb titres over time. We included all individuals with a complete sample series who were nAb positive within the study timeframe, and grouped them by exposure type (infected, vaccinated, and infected and vaccinated), and survey of seroconversion. A large degree of heterogeneity in the nAb responses was observed, with both the magnitude of boosting and waning varying substantially between participants (Supplementary Fig. 3). Additionally, within-individual responses varied greatly between surveys, though as observed with Fig. 2 those seroconverted prior to Survey 1 had more consistent titres. When comparing the responses to different variants, there was a high degree of cross-reactivity – for example, individuals infected at Survey 4 were most likely infected with an Omicron variant, but also displayed responses to the other SARS-CoV-2 variants. Notably, seroconversions at Survey 3 were dominated by those vaccinated, with many then becoming infected and vaccinated at Survey 4. In contrast, Survey 4 seroconversions were dominated by infections.

### Vaccination leads to higher nAb responses than infection

**Fig. 3:**
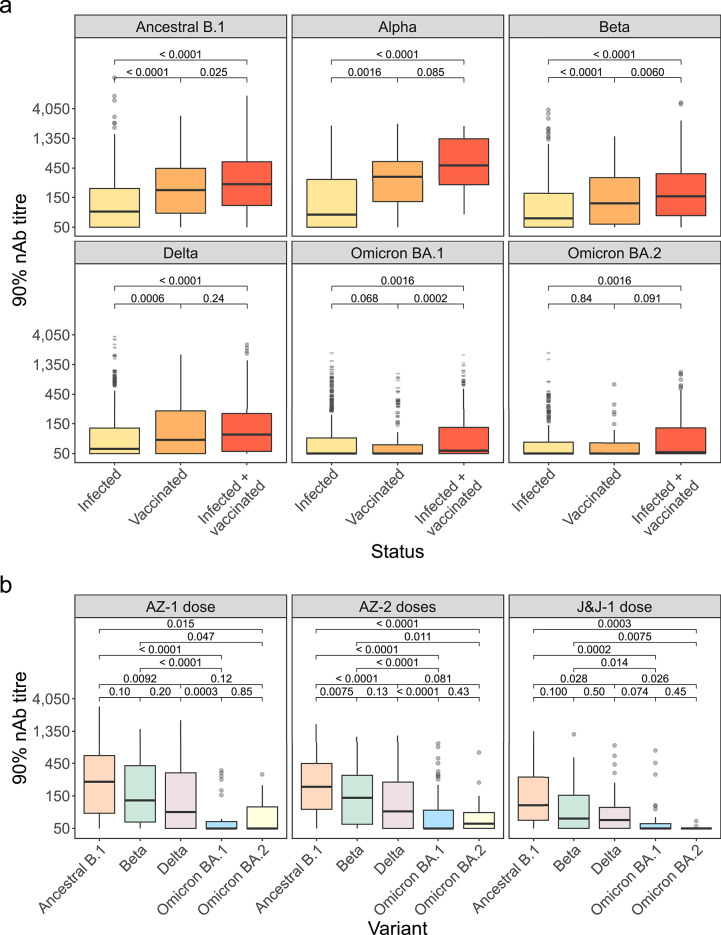
Association between SARS-CoV-2 neutralising antibody (nAb) titres and SARS-CoV-2 exposure history, as well as between nAb titres and the SARS-CoV-2 variant targeted, among vaccinated individuals. (**a**) Boxplots comparing nAb titres among those with different SARS-CoV-2 exposure types: infected only (yellow), vaccinated only (orange), and both infected and vaccinated (red) (total *n* = 734 SARS-CoV-2 nAb positive samples from participants across the study surveys, infected – *n* = 500, vaccinated – *n* = 134, infected + vaccinated – *n* = 210) Infection history among vaccinated individuals was determined using a Nucleocapsid ELISA. (**b**) Boxplots comparing nAb titres against different SARS-CoV-2 variants (colours as in Fig. 1 sankey plot), stratified by COVID-19 vaccine type (AstraZeneca (AZ)-one dose, *n* = 50; AZ-two doses, *n* = 72; and Johnson & Johnson (J&J)-one dose, *n* = 12) (total *n* = 134 – samples from vaccinated participants who were positive for neutralising antibodies, study surveys combined). Titres were measured using HIV(SARS-CoV-2) PVNA. Boxplots display the median and interquartile range (IQR) of the outcome (90% titre). Statistical test used was Wilcoxon rank sum test (Benjamini-Hochberg (BH) adjustment), with the p-value for the relationship between different groups displayed.

To examine the strength of participants’ protective, neutralisation responses, we focused on the titrated samples. We compared nAb titres in those solely infected, solely vaccinated, and both infected and vaccinated. Individuals both infected and vaccinated had higher nAb responses than those infected for all variants (ancestral B.1, *p* < 0.0001; Alpha, *p* < 0.0001; Beta, *p* < 0.0001; Delta, *p* < 0.0001; Omicron BA.1, *p* = 0.0016; Omicron BA.2, *p* = 0.0016; Wilcoxon) (Fig. 3A). For ancestral B.1, Alpha, Beta, and Delta, the median nAb titres for those vaccinated were significantly higher compared with those infected (ancestral B.1: 196.7, IQR 84.1-447.3 vs. 89.1, IQR 50.0-209.1, respectively, *p* < 0.0001; Alpha: 332.7, IQR 129.3-576.1 vs. 79.9, IQR 50.0-297.0, *p* = 0.0016; Beta: 121.4, IQR 56.1-312.4 vs. 69.2, IQR 50.0-175.2, *p* < 0.0001; Delta: 82.5, IQR 50.0-239.9 vs. 59.4, IQR 50.0-127.9, *p* = 0.0006; Wilcoxon) (Fig. 3A).

To study vaccination-induced nAbs solely, we analysed vaccinated (not infected) individuals. Alpha was excluded as it was only tested against at Survey 1 when participants had received just AZ-one dose. Vaccinated individuals displayed their highest nAb activity against ancestral B.1 (AZ-one dose: 240.3, IQR 83.6-586.6; AZ-two doses: 203.8, IQR 95.1-449.1; J&J-one dose: 109.1, IQR 65.8-282.2; Wilcoxon) (Fig. 3B). Neutralisation was lower against other variants, decreasing as variants diverged from the ancestral B.1 virus. Omicron BA.1 and BA.2 were poorly neutralised (AZ-one dose, BA.1: 50.0, IQR 50.0–63.0; BA.2: 50.0, IQR 50.0-132.0) (AZ-two doses, BA.1: 50.0, IQR 50.0-92.8; BA.2: 59.6, IQR 50.0-85.5) (J&J-one dose, BA.1: 50.0, IQR 50.0-58.6; BA.2: 50.0, IQR 50.0–50.0) (Fig. 3B). Next, nAb responses generated by different vaccination types were assessed. No differences in SARS-CoV-2 nAb titres were detected when comparing individuals who had received AZ-one dose, AZ-two doses, and J&J-one dose (Supplementary Fig. 4).

### Weaker nAb responses in younger participants (< 15 years) post infection

**Fig. 4:**
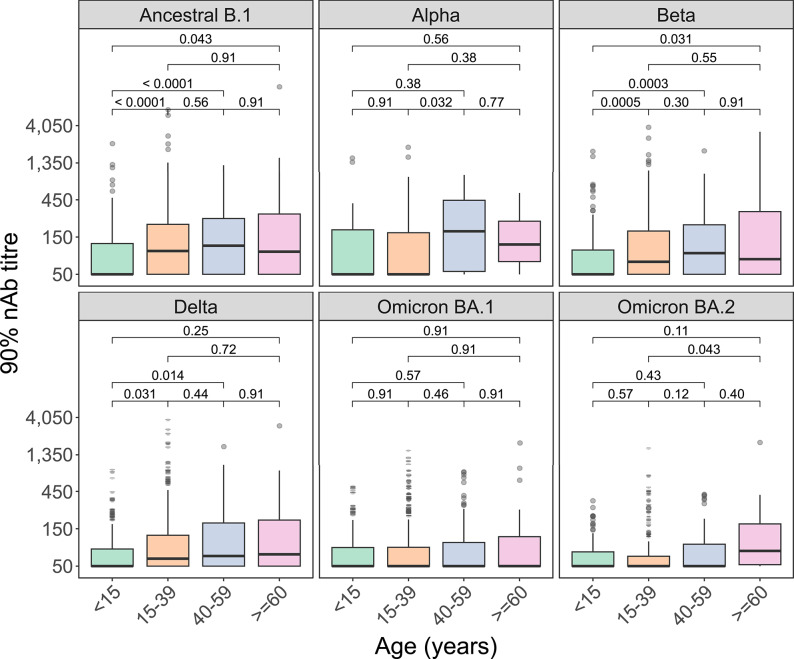
Association between SARS-CoV-2 neutralising antibody (nAb) titre and age at sample collection – post SARS-CoV-2 infection (not vaccination). SARS-CoV-2 nAb titres in those SARS-CoV-2 infected only were partitioned based on age category (participants aged *≥* one year) and SARS-CoV-2 variant titred against (*n* = 500 unvaccinated, SARS-CoV-2 nAb positive samples from participants across the study surveys). Titres were measured using HIV(SARS-CoV-2) PVNA. Boxplots display the median and interquartile range (IQR) of the outcome (90% titre). Those < 15 years shown in green (*n* = 129), 15–39 years in orange (*n* = 265), 40–59 years in blue (*n* = 83), and > = 60 in pink (*n* = 23). Statistical test used was Wilcoxon rank sum test (Benjamini-Hochberg (BH) adjustment), with the p-value for the relationship between different groups displayed.

Among SARS-CoV-2 infected (not vaccinated) participants, those aged < 15 years had lower nAb titres, indicating lower protective immunity, to ancestral B.1 and Beta compared with all older age categories (For B.1, < 15 years: nAb titre of 50.0, IQR 50.0-123.8 vs. 15–39 years: 100.0, IQR 50.0-218.7, *p* < 0.0001; 40–59 years: 116.3, IQR 50.0-259.1, *p* < 0.0001; ≥60 years: 97.6, IQR 50.0-298.4, *p* = 0.043; Wilcoxon) (For Beta, < 15 years: nAb titre of 50.0, IQR 50.0-102.2 vs. 15–39 years: 72.3, IQR 50.0-179.0, *p* = 0.0005; 40–59 years: 93.3, IQR 50.0-215.7, *p* = 0.0003; ≥60 years: 78.3, IQR 50.0-323.6, *p* = 0.031; Wilcoxon) (Fig. 4). For Delta, those < 15 years (50.0, IQR 50.0-82.7) had lower nAb responses than those 15–39 years (62.5, IQR 50.0-124.5, *p* = 0.031) and 40–59 years (67.5, IQR 50.0-177.8, *p* = 0.014; Wilcoxon). Among those vaccinated (not infected) (≥15 years only), no differences by age group were observed (Supplementary Fig. 5). For those infected and vaccinated, the 15–39 year olds had lower nAb titres than the 40–59 year olds (for ancestral B.1, *p* = 0.044; Beta, *p* = 0.0036; Delta, *p* = 0.044; Wilcoxon) and the ≥60 year olds (for ancestral B.1, *p* = 0.012; Beta, *p* = 0.0036; Delta, *p* = 0.044; and Omicron BA.2, *p* = 0.044; Wilcoxon) (Supplementary Fig. 5).

### Comorbidity reporting did not influence nAb titres

We determined whether comorbidity status influenced SARS-CoV-2 nAb titres. Among SARS-CoV-2 infected (not vaccinated) participants we observed no significant difference between those reporting no comorbidities vs. those reporting at least one comorbidity (for ancestral B.1, *p* = 0.89; Alpha, *p* = 0.97, Beta, *p* = 0.65; Delta, *p* = 0.65; Omicron BA.1, *p* = 0.97; Omicron BA.2, *p* = 0.69, Wilcoxon) (Supplementary Fig. 6). The same was observed among vaccinated participants (combining both those infected and vaccinated and solely vaccinated under “COVID-19 vaccinated” due to limited participants when stratifying) (Supplementary Fig. 6).

### Lower nAb titres in HIV-infected individuals than HIV-uninfected individuals

**Fig. 5:**
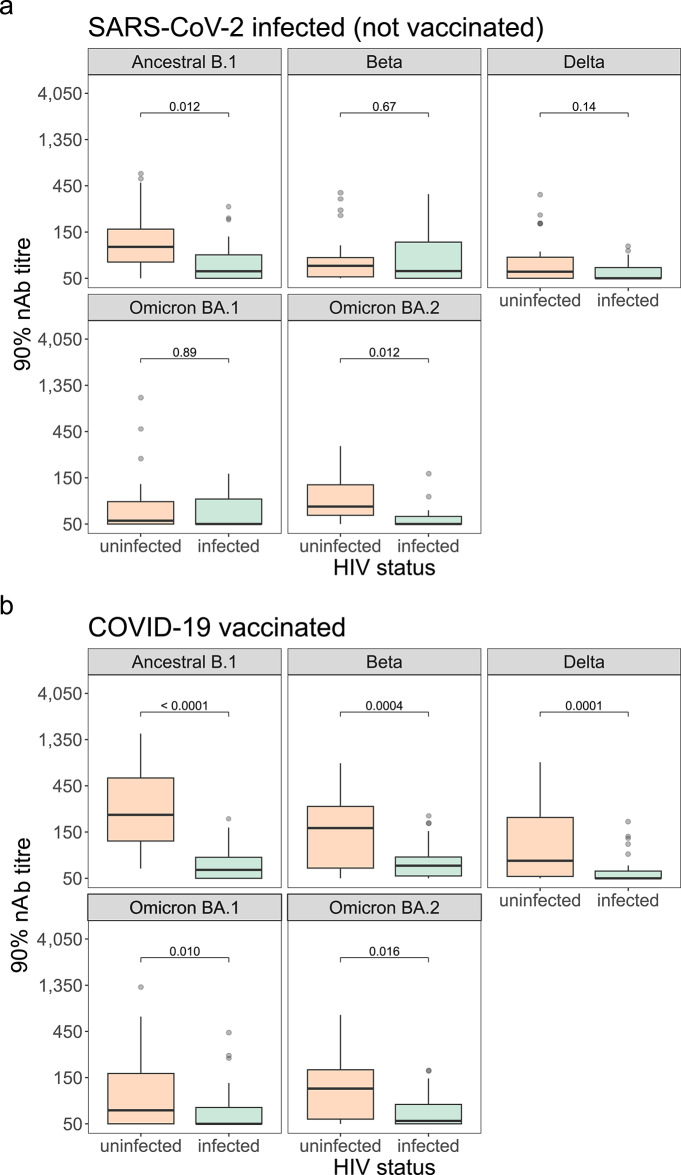
Effect of HIV infection status on SARS-CoV-2 neutralising antibody (nAb) titre using VSV(SARS-CoV-2) PVNA. (**a**) Boxplots comparing SARS-CoV-2 nAb titres to different variants in HIV-uninfected (*n* = 26, orange) and HIV-infected serum samples (*n* = 24, green). This includes samples from participants who have been SARS-CoV-2 infected (not COVID-19 vaccinated) and were nAb positive, study surveys combined. (**b**) Boxplots comparing nAb titres to different SARS-CoV-2 variants in HIV-uninfected (*n* = 31 surveys combined, orange) and HIV-infected (*n* = 45 surveys combined, green). This includes samples from participants who were COVID-19 vaccinated (those SARS-CoV-2 infected and vaccinated, and vaccinated (not infected)) and were nAb positive, study surveys combined. Samples were categorised by HIV infection status and SARS-CoV-2 variant titred against. Box plots display the median and interquartile range (IQR) of the outcome (90% titre). Statistical test used was the Wilcoxon rank sum test (Benjamini-Hochberg (BH) adjustment), with the p-value for the relationship between different groups displayed.

The effect of HIV status on nAb titres was investigated using the VSV-based pseudotype system, comparing all HIV-infected participants with a representative subset of HIV-uninfected participants that were tested with this assay. As above, due to limited samples from people living with HIV, those infected and vaccinated, and solely vaccinated were combined under “COVID-19 vaccinated”. Alpha was again excluded as it was only tested at Survey 1, when few HIV-infected participants (*n* = 4) were SARS-CoV-2 nAb positive. Among SARS-CoV-2 infected (not vaccinated) individuals, people living with HIV had lower nAb titres to ancestral B.1 and Omicron BA.2 than HIV-uninfected participants (ancestral B.1: 59.1, IQR 50.0-88.6 vs. 105.6, IQR 73.6-161.1, respectively, *p* = 0.012; Omicron BA.2: 50.0, IQR 50.0–60.0 vs. 75.8, IQR 61.6-130.9, *p* = 0.012; Wilcoxon) (54). No significant differences were observed for Beta, Delta, and Omicron BA.1 (Beta, *p* = 0.67; Delta, *p* = 0.14; Omicron BA.1, *p* = 0.89) (Fig. 5). Among COVID-19 vaccinated individuals, those living with HIV had lower nAb titres across variants compared with HIV-uninfected participants (ancestral B.1: 61.1, IQR 50.0-82.4 vs. 225.8, IQR 121.1-542.5, respectively, *p* < 0.0001; Beta: 67.5, IQR 52.9–82.9 vs. 164.8, IQR 63.8-275.8, *p* = 0.0004; Delta: 50.0, IQR 50.0-59.3 vs. 75.9, IQR 52.3-212.9, *p* = 0.0001; Omicron BA.1: 50.0, IQR 50.0-73.7 vs. 68.9, IQR 50.0-165.4, *p* = 0.010; Omicron BA.2: 53.8, IQR 50.0-79.4 vs. 115.6, IQR 56.0-184.6, *p* = 0.016; Wilcoxon) (Fig. 5).

## Discussion

This longitudinal community-based serosurveillance study provides a comprehensive understanding of SARS-CoV-2 protective immunity in both rural and urban regions of Malawi. By tracking nAb responses over time, the study captures the evolving landscape of variant-driven infections and the impact of increasing vaccination coverage. Early infections were primarily driven by ancestral B.1 and Beta variants, while Delta and Omicron BA.1/BA.2 dominated later study surveys, reflecting the documented transmission waves in Malawi^6^.

Individuals with “Delta-dominant” immune responses frequently reverted to seronegative status, suggesting limited sustained protective immunity post-Delta infection. This finding has important implications for regions where Delta was prevalent and underscores the potential need for post-infection vaccination to enhance long-term immunity. It should be noted that a previous study detected relatively high levels of seroreversion, with 15.4% of participants in this study having no detectable nAbs by 3-months post exposure^14^.

Individuals in Lilongwe (the urban site) displayed higher infection rates, consistent with global trends of increased transmission in cities^15^. Additionally, Lilongwe’s status as a transportation hub likely contributed to increased viral introduction and spread. Vaccination rates were also higher in Lilongwe - rural Malawian regions have limited access to COVID-19 vaccination and greater vaccine hesitancy, explaining the lower uptake in Karonga^16,17^. By Survey 4, rates of hybrid immunity were high, with 73% of vaccinated participants testing N ELISA positive. This reflects the growing complexity of SARS-CoV-2 exposure histories, as a high proportion of individuals had experienced both vaccination and infection.

Participants who were seropositive at Survey 1, (infected during the ancestral B.1 or Beta waves) on average had stronger neutralising responses, particularly following Omicron reinfection - possibly due to immune imprinting. While this phenomenon has been described elsewhere^18^, our finding is novel but requires cautious interpretation given the small number of early seropositive participants in the cohort. However, the high magnitude of boosting in Survey 1-seroconverted participants on reinfection suggests that their prior exposure primed the body to respond to future SARS-CoV-2 variants very effectively. Analysis of individual-level responses over time revealed substantial heterogeneity both between participants and within participants across study surveys, highlighting the dynamic nature of nAb responses and the challenges in their interpretation.

Consistent with prior research^19^, vaccinated individuals—whether previously infected or not—exhibited stronger neutralising responses than those infected only. We observed no significant differences by vaccination type, potentially due to limited vaccinated participants resulting in reduced statistical power, or because the vaccines administered to the participants were adenoviral vector-based, which may elicit similar nAb responses. Nonetheless, other studies identified weaker responses post J&J vaccination due to the single dose regimen^20^.

Participants < 15 years old had low nAb titres post-infection (but not post-vaccination) making them more susceptible to reinfection. Other research has agreed^21^, while some reported similar neutralising responses in both children and adults^22^, as well as a potential decrease in neutralising activity with age^23^. Our study potentially lacked power to assess differences in adults ≥60 years due to small numbers.

No differences in nAb titres by comorbidity status were found, agreeing with other research^24,25^. Comparisons by self-reported HIV status among SARS-CoV-2 infected (not vaccinated) participants showed that for the ancestral B.1 virus and Omicron BA.2 variant, those HIV-infected had lower nAb responses than those HIV-uninfected. However, statistical differences were not observed for Beta, Delta, and Omicron BA.1 likely reflecting a combination of low statistical power due to relatively small sample sizes and the lower levels of neutralisation observed for these variants. Median titres for these variants still trended higher in HIV-uninfected participants, suggesting that a true difference may exist but was not detectable. In contrast, for ancestral B.1 and Omicron BA.2, the higher nAb responses resulted in a larger magnitude of between-group differences, allowed statistical significance to be reached. The stronger ancestral B.1 nAb responses observed was consistent with prior reports that the ancestral virus elicits higher nAb immune responses^26^. For Omicron BA.2, the higher titres likely reflect the timing of sample collection, as many nAb-positive samples were obtained during Survey 4, coinciding with the emergence of Omicron BA.2 in the population, suggesting recent infection with this variant in some participants.

When looking among those COVID-19 vaccinated, HIV-uninfected individuals had lower nAb responses than those HIV-infected for all variants tested against. Given that neutralising responses are indicative of protective immunity^13^, this suggests that people living with HIV are more vulnerable to reinfection. Conflictingly, other research reports no differences when comparing SARS-CoV-2 nAbs in HIV-infected and HIV-uninfected participants^27^. Their study, however, focused on HIV-infected individuals receiving antiretroviral therapy (ART) and did not include ART-naïve HIV-infected persons. Another study where HIV-infected individuals with poorly controlled HIV were included observed lower nAb levels in HIV-infected participants^28^. Our study included people living with HIV who were not receiving ART, however the limited number of these individuals (*n* = 10) precluded comparisons by ART status due to lack of statistical power. HIV status and ART receipt were self-reported; we previously demonstrated that the latter was inaccurate on occasion^29^. Additionally, CD4 data were not obtained as detailed HIV characterisation was not a study objective, and the cost of this test made it infeasible given the large sample size and budget constraints. We therefore are unable to account for the degree of HIV-associated immunosuppression in these participants.

Our findings reaffirm the role of vaccination in generating robust protective immunity. In low-resource settings with limited vaccine supply and vaccine hesitancy prevalent, both targeted vaccination of at-risk groups and efforts to motivate vaccination are key. Although younger participants had lower nAb titres, vaccination is considered unnecessary for this demographic due to their typically milder disease outcomes. However, their reduced nAb levels may result in increased risk of breakthrough infections. We therefore recommend targeted surveillance of transmission within this demographic to mitigate potential spread. We recommend prioritising vaccination of high-risk groups for severe disease, especially those living with HIV. HIV-infected individuals generated poor neutralising responses post vaccination, necessitating booster doses. If possible, these individuals should receive mRNA COVID-19 vaccines which induce stronger nAb responses than the adenoviral vector vaccines^26^. The Pfizer-BioNTech (Comirnaty) COVID-19 mRNA vaccine was introduced in Malawi in January 2022, targeting individuals aged 12–17 years. Newer COVID-19 mRNA vaccines can be stored at higher temperatures, easing use in low-resource settings. Additionally, future vaccination campaigns should prioritise use of vaccines updated for recent variants, such as JN.1-derivatives.

Strengths of our study include its longitudinal design, random household sampling, and the inclusion of both children and people living with HIV. Importantly, we used neutralisation assays to infer prior variant exposure, a useful proxy in settings with limited genomic surveillance capacity. However, the study was not without its limitations. The high cut-off threshold (90%) for defining neutralisation potentially overlooked low-level responses and may have contributed to the relatively high levels of seroreversion observed between surveys. The assigning of a dominant variant per participant does not specify the infecting variant as this is unknown. Instead, it characterises the variant-specific immune response measured in the participants when considering that multiple viral variants are cocirculating. As mentioned above, the collection of HIV related data was limited in that HIV status and ART receipt were self-reported and CD4 counts were not determined. Another limitation is the inability to incorporate “time since exposure” as a covariate. For these participants, the timing of their most recent exposure is unknown due to the lack of routine diagnostic testing. Ongoing work is focused on modelling individual infection histories within this cohort, which will allow us to estimate the timing of SARS-CoV-2 infections and the subsequent waning of antibodies, even in the context of limited diagnostic testing.

In summary, this study provides critical insights into the shifting dynamics of SARS-CoV-2 immunity in Malawi. We concluded that the prevalence SARS-CoV-2 protective immunity increased over the study period with increasing exposures to emerging variants and rising vaccination rates. We observed a higher prevalence in the urban site compared with the rural site, with the strength of neutralisation being influenced by the variant people have been exposed to, vaccination, age, and HIV status. These findings underscore the need for targeted public health interventions—particularly improved vaccine accessibility, prioritisation of high-risk groups, and continued surveillance—to reduce the burden of COVID-19 in resource-limited settings. In particular, enhanced monitoring of children and individuals living with HIV is encouraged, given their elevated risk of breakthrough infections. Focused vaccination strategies for people living with HIV, including access to more immunogenic vaccine platforms and booster doses, should be prioritised.

## Methods

### Participants

The cohort included individuals aged ≥ one year from randomly selected households involved in population cohorts, operated by the Malawi Epidemiology and Intervention Research Unit (MEIRU) (*n* = 2,005): rural site - Karonga Health Demographic Surveillance Site; urban site - Area 25, Lilongwe (Supplementary Fig. 7). Participants were recruited from 24th February-8th June 2021 (Survey 1) (Supplementary Fig. 8). They were subsequently surveyed three times at three-month intervals – Survey 2: 28th June-13th September 2021; Survey 3: 4th October-10th December 2021; Survey 4: 27th January-22nd April 2022 (Supplementary Fig. 8). Households invited to participate in the study were selected using a randomised spatial inhibitory design, details in Banda et al.^6^. Written, informed consent was obtained from participants or their guardians (if < 15 years old or a vulnerable adult).

### Sample and metadata collection

An interview-led questionnaire collected baseline demographics (including self-reported age and biological sex assigned at birth); socioeconomic indicators; medical history (including self-reported HIV status); COVID-19 symptoms; COVID-19 vaccination history; and infection preventative behaviours. Subsequent surveys collected updated information on COVID-19 vaccination, symptoms, and any healthcare received. Venous blood was collected at each survey, with serum stored at MEIRU (-80 °C) before shipment to the MRC-University of Glasgow Centre for Virus Research. This analysis included all adults plus 50% of the children (due to limited blood volume collection)-1,876/2,005 participants (Survey 1: *n* = 1,515; Survey 2: *n* = 1,322; Survey 3: *n* = 1,215; Survey 4: *n* = 1,123 – participants lost to follow-up) (Supplementary Fig. 9).

### Cells

Cells were maintained at 37 °C, 5% CO_2_. HEK293-ACE2 cells (produced by stable transduction of HEK293 cells with pSCRPSY-hACE2 which encodes the human ACE-2 receptor) were gifted by Matt Turnball and Suzannah Rihn (MRC-University of Glasgow Centre for Virus Research). These cells were maintained in Dulbecco’s Modified Eagle’s Medium (DMEM, Gibco) with 10% foetal bovine serum (Gibco), 2 mM L-glutamine (Gibco), 100 µg/ml streptomycin (Gibco), and 100 IU/ml penicillin added (Gibco) (complete DMEM), supplemented with 2 µg/ml puromycin. HEK293T cells (from American Type Culture Collection) were maintained in complete DMEM supplemented with 400 µg/ml G418.

### SARS-CoV-2 serology

Two different PVNA systems were used. For self-reported HIV-uninfected participants, an HIV-based assay system was used to measure SARS-CoV-2 neutralising antibodies (nAbs). For HIV-infected participants, and a representative subset of HIV-uninfected participants for comparison, a vesicular stomatitis virus (VSV)-based assay was used instead. This was necessary because integrase inhibitors, commonly taken by people living with HIV, interfere with the HIV-based pseudotyping vector and reduce nAb measurement accuracy^29^. The VSV-based system avoids this interference because, as a rhabdovirus, it is not affected by integrase inhibitors. Despite this limitation for HIV-infected samples, the HIV-based assay was retained for HIV-uninfected participants because it has shown better agreement with live virus assays than the VSV-based system for these participants, particularly at low and high percent neutralisation levels^29^.

#### HIV-based SARS-CoV-2 PVNA

HIV-based SARS-CoV-2 pseudotypes were generated (Supplementary Methods) and a PVNA was performed on self-reported HIV-uninfected participants, as described^13^. Initially, samples were tested in a single dilution screen for neutralising activity against ancestral B.1 virus, and the Beta, Delta, and Omicron BA.1 variants (Supplementary Table 1). Serum samples and controls were diluted 1 in 25 in complete DMEM and added (25 µl per well) in duplicate to white 96 well plates before incubation at 37 °C for one hour with the specified pseudovirus variant (25 µl per well). HEK293-ACE2 target cells (diluted to 4 × 10^5^ cells/ml, 50 µl per well) were added and incubated for 48–72 h at 37 °C. Luciferase activity was measured by adding Steadylite Plus chemiluminescence substrate diluted (75 µl, diluted 1 in 3, Revvity, Beaconsfield, UK), and analysed with an EnSight multimode plate reader (Revvity, Beaconsfield, UK). Samples showing percent neutralisation > 90% were classed as positive. Controls included a no serum control (complete DMEM), positive control (pooled SARS-CoV-2 positive sera collected from March to May 2020, ethics provided by the NHS Greater Glasgow and Clyde (GGC) Biorepository (application 550)), and a negative control (pre-pandemic serum sample from a single individual, ethics provided by the Scottish National Blood Transfusion Service protocol NATF 765 10).

Positive samples were titrated against specific SARS-CoV-2 variants, based on those thought to have circulated in Malawi at each study survey^6^: Survey 1 – ancestral B.1, Alpha, Beta, Delta; Survey 2 – ancestral B.1, Beta, Delta, Omicron BA.1; Survey 3 – ancestral B.1, Beta, Delta, Omicron BA.1; Survey 4 – ancestral B.1, Beta, Delta, Omicron BA.1, Omicron BA.2 (Supplementary Table 1). Titrations were performed by serially diluting the serum samples in complete DMEM – 3-fold dilutions from 1 in 25 to 1 in 18,225. The diluted sera was added to the plate (25 µl in triplicate) before incubation with the virus (25 µl per well) for one hour at 37 °C. HEK293-ACE2 target cells were added (diluted to 4 × 10^5^ cells/ml, 50 µl per well) and incubated for 38–72 h at 37 °C. Luciferase activity was measured by adding Steadylite Plus chemiluminescence substrate (75 µl diluted 1 in 3, Revvity, Beaconsfield, UK) and analysed with a EnSight multimode plate reader (Revvity, Beaconsfield, UK). Titres were calculated at 90% reduction in infectivity. A participant’s dominant variant was identified by the variant against which samples displayed the highest titre. If two variants had no significant difference, both were listed. If there was no difference between > 2 variants, the response was categorised as “broad”. Samples with ancestral B.1/Alpha responses were labelled ancestral B.1 as Alpha circulation was not reported in Malawi.

#### VSV-based SARS-CoV-2 PVNA

VSV-based SARS-CoV-2 pseudotypes were generated (Supplementary Methods) and used to screen self-reported HIV-infected participant serum samples (*n* = 96) and a random selection of HIV-uninfected participants (*n* = 180) for SARS-CoV-2 nAbs. These assays were performed as above for HIV(SARS-CoV-2) PVNA, with the same controls included.

#### Nucleocapsid ELISA

Sera from vaccinated participants were tested for IgG antibodies against the SARS-CoV-2 nucleocapsid (N) protein (Wuhan-Hu-1, GenBank: MN908947) using an ELISA^13^. The ELISA distinguished “vaccinated (not infected)” participants from “infected and vaccinated”. Briefly, 96 well plates were coated with N protein (50 µl per well) that had been diluted to 1.0 µg/ml in phosphate buffered saline (PBS, Gibco) and incubated overnight at 4 °C. Plates were washed three times with phosphate-buffer solution (PBS/0.05%Tween20) - additional wash steps followed this protocol. Blocking buffer (PBS/0.05%Tween20 with 10% casein (Vector laboratories, 2BScientific, Upper Heyford, UK), 200 µl) was added. Plates were incubated for one hour before a second washing step. Diluted sera and controls (1 in 100 in blocking buffer, 50 µl per well) were added in duplicate and incubated for one hour before being washed. Anti-human IgG horseradish peroxidase-conjugated secondary antibody (Bethyl laboratories, Cambridge Bioscience, Cambridge, UK) was diluted 1 in 3,000 in blocking buffer and added (50 µl per well). Plates were incubated for one hour and then washed. Following this, 3,3′,5,5′-tetramethylbenzidine was added (50 µl per well, KPL, Gaithersburg, USA) and the plates were incubated for 10 min in the dark. The reaction was stopped by adding 1-mol/L sulfuric acid (50 µl per well) and plates were read at 450 nm using a Multiskan FC plate reader Versa (Thermofisher Scientific, UK). Samples with a normalised absorbance ≥ 3.666 were considered positive. Control samples were the same as above for the HIV(SARS-CoV-2) single dilution screening.

### Data analyses

Data were analysed using RStudio (version 2024.12.0 + 467)^30^. Longitudinal analyses of participants over the four study surveys (Figs. 1 and 2, Supplementary Fig. 3) are restricted to participants who contributed a complete sample series. Sankey plots that display the SARS-CoV-2 neutralising responses over time were created with the networkD3 package, version 0.4^31^. Other analyses and visualisations were performed using ggplot2 (version 3.5.1)^32^ and ggpubr (version 0.6.0)^33^.

For participant characteristics, when comparing continuous variables between groups, the Wilcoxon rank-sum test was used as the variables were not normally distributed. Categorical variables were analysed using either the chi-squared test or Fisher’s exact test. The chi-squared test was used when cell frequencies were ≥ 5. When one or more cell counts were < 5, Fisher’s exact test was applied as it provides more accurate results under these conditions. For seroprevalence estimates, 95% binomial confidence intervals (CIs) were reported, and group differences were assessed using two-proportion chi-squared tests (two-tailed), appropriate when comparing categorical proportions with sufficient sample sizes (*n* ≥ 5). Antibody titres were summarised using the median and interquartile range (IQR) of 90% neutralising antibody titres. Group differences were evaluated using the Wilcoxon rank-sum test (two-tailed), a non-parametric method suitable for continuous data that are not normally distributed. Normality was assessed using visual methods, including histograms and boxplots to examine the distribution of data. A false discovery rate correction using the Benjamini-Hochberg (BH) method was applied to reduce Type 1 errors when performing multiple comparisons. An alpha level of 0.05 was used to determine statistical significance. P-values were reported as exact values, except when very small, in which case they are presented as *p* < 0.0001. Missing data (i.e., from participant dropouts) were handled as ‘not applicable’ (‘NA’). Cohort size calculation is described in the Supplementary Methods.

## Supplementary Information

Below is the link to the electronic supplementary material.

Supplementary Material 1

## Data Availability

The data that support the findings of this study are available from the Malawi Epidemiology and Intervention Research Unit (MEIRU) but restrictions apply to the availability of these data, which were used under license for the current study, and so are not publicly available. Data are however available from the authors upon reasonable request and with permission of MEIRU.
