# Supplementary material for: Characterising protective immune responses to SARS-CoV-2 in urban and rural Malawi between February 2021 and April 2022
Source: Sci Rep. 2025 Oct 29;15:37914. doi: 10.1038/s41598-025-22599-7 (PMC12572322; doi:10.1038/s41598-025-22599-7)
Supplement: Supplementary file 1 — Supplementary Material 1 [file 41598_2025_22599_MOESM1_ESM.pdf]

Characterising protective immune responses to SARS-CoV-2 in urban and rural Malawi between February 2021 and April 2022

Authors:

Mhairi J. McCormack BSc (Hons)[1] - m.mccormack.1@research.gla.ac.uk
Louis Banda MSc[2*] - louis.banda@meiru.mw
Stephen Kasenda MBBS[2*] - stephen.kasenda@meiru.mw
Ellen C. Hughes PhD[1,3] – ellen.hughes@liverpool.ac.uk
Lina Leonhard MSc[1] – lina.leonhard@yahoo.de
Annie Mwale MPH[4] – chaumaannie@gmail.com
Estelle McLean PhD[2] – estelle.mclean@yahoo.com
Alison Price PhD[2] – alison.price@lshtm.ac.uk
Amelia Crampin MSc[2,5,6] – mia.crampin@lshtm.ac.uk
David Chaima PhD[7] – dchaima@kuhes.ac.mw
Abena S. Amoah PhD[2,8†] - a.s.amoah@lumc.nl
Tonney S. Nyirenda PhD[7†] - tnyirenda@kuhes.ac.mw
Antonia Ho PhD[1†] - Antonia.Ho@glasgow.ac.uk
Brian J. Willett PhD[1†] - Brian.Willett@glasgow.ac.uk

*These authors contributed equally
†These authors jointly supervised this work

Affiliations:

1. Medical Research Council-University of Glasgow Centre for Virus Research, Glasgow, UK
2. Malawi Epidemiology and Intervention Research Unit (MEIRU), Malawi
3. Department of Livestock and One Health, Institute of infection, veterinary and ecological sciences, University of Liverpool, Liverpool, UK
4. Public Health Institute of Malawi, Lilongwe, Malawi
5. London School of Hygiene and Tropical Medicine, London, UK
6. School of Health and Wellbeing, University of Glasgow, Glasgow, UK
7. Kamuzu University of Health Sciences (KUHeS), Blantyre, Malawi
8. Leiden University Medical Center, Leiden, Netherlands

Contents:

Supplementary Methods 3

Generation of HIV(SARS-CoV-2) pseudotypes 3
Generation of VSV(SARS-CoV-2) pseudotypes 3
Sample size calculation 3

Supplementary Tables 4

Supplementary Table 1. SARS-CoV-2 Spike gene construct mutations, relative to the Wuhan-Hu-1 sequence (GenBank: MN908947). 4

Supplementary Figures 5

Supplementary Figure 1. COVID-19 vaccination coverage in Karonga and Lilongwe across the four study surveys (n=1,876). 5
Supplementary Figure 2. SARS-CoV-2 neutralising antibody (nAb) prevalence in individuals from Karonga (orange) and Lilongwe (green) across the four study surveys. 6
Supplementary Figure 3. Longitudinal neutralising antibody (nAb) trajectories per participant. 7
Supplementary Figure 4. Neutralising antibody titres by COVID-19 vaccination type 8
Supplementary Figure 5. Association between SARS-CoV-2 neutralising antibody (nAb) titre and age at sample collection. 10
Supplementary Figure 6: SARS-CoV-2 neutralising antibody (nAb) titre by comorbidity status. 12
Supplementary Figure 7. Map of Malawi, showing the location of the Karonga Health Demographic Surveillance Site (HDSS) and Area 25, Lilongwe. 12
Supplementary Figure 8. Survey periods for the study. 13
Supplementary Figure 9. Flow chart of study participants. 14

Supplementary References 14

Supplementary Methods

Generation of HIV(SARS-CoV-2) pseudotypes

HEK293T cells were transfected with the appropriate SARS-CoV-2 spike gene expression vector (ancestral B.1, Alpha, Beta, Delta, Omicron BA.1, Omicron BA.2 – 0.15 µg/ml) together with p8.91[1] (0.1 µg/ml, HIV gag-pol plasmid from Addgene) and pCSFLW[2] (0.1 µg/ml, firefly luciferase plasmid) using 1µl/ml polyethylenimine transfection agent (PEI, Polysciences, Warrington, USA). Supernatants containing HIV(SARS-CoV-2) pseudotypes were harvested 48 hours post-transfection, passed through a 0.45 µm filter, aliquoted, and stored at −80°C. Gene constructs bearing the ancestral B.1 virus (D614G), Alpha (B.1.1.7), Beta (B.1.351), Delta (B.1.617.2), Omicron BA.1 (B.1.1.529) and Omicron BA.2 spike genes were based on the codon-optimised spike sequence of SARS-CoV-2 and generated by GenScript Biotech (Supplementary Table 1).

Generation of VSV(SARS-CoV-2) pseudotypes

To generate the VSV(SARS-CoV-2) pseudoviruses, the appropriate SARS-CoV-2 spike gene expression vectors (ancestral B.1, Alpha, Beta, Delta, Omicron BA.1, Omicron BA.2 – 0.5 µg/ml) were combined with polyethylenimine (PEI, Polysciences, Warrington, USA, 6µl/ml), added to HEK293T cells and incubated for four hours at 37°C. VSV-DG luc (containing the VSV genes - excluding the G protein – and the firefly reporter gene; kindly gifted from Michael Whitt, Memphis, Tennessee, USA) was added to the cells at a multiplicity of infection (MOI) of 0.02 and incubated for one hour at 37°C. The medium was removed, and the cells were washed three times with phosphate buffered saline (PBS, Gibco). Complete DMEM was added, and the cells were incubated for 48-hours at 37°C. Pseudoviruses were harvested by passing the supernatant through a 0.45 µm filter, aliquoted and stored at -80°C.

Sample size calculation

Sample size was calculated, as described previously[3]. Briefly, we assessed the study's power to detect differences in antibody prevalence between sampling strata, assuming 1,000 individuals per stratum (e.g., per urban/rural study site). The detectable difference depends on the true prevalence in each group (sample A/sample B). If the true prevalence in sample A is 5%, the study has 80% power to detect a sample B prevalence of <2.4% or >8.2% (p<0.05 significance level). If the true prevalence of sample A is 10%, the study has 80% power to detect prevalence <6.3% or >14.4% in sample B.

Supplementary Tables

Supplementary Table 1. SARS-CoV-2 Spike gene construct mutations, relative to the Wuhan-Hu-1 sequence (GenBank: MN908947).

Variant	Mutations relative to Wuhan-Hu-1 sequence
Ancestral (B.1)	D614G
Alpha (B.1.1.7)	Δ69–70, Δ144, N501Y, A570D, D614G, P681H, T716I, S982A, D1118H
Beta (B.1.351)	D80A, D215G, L241del, L242del, A243del, K417N, E484K, N501Y, D614G, A701V
Delta (B.1.617.2)	T19R, G142D, Δ156-157, R158G, L452R, T478K, D614G, P681R, D950N
Omicron BA.1 (B.1.1.529)	A67V, Δ69–70, T95I, G142D/Δ143–145, Δ211/L212I, ins214EPE, G339D, S371L, S373P, S375F, K417N, N440K, G446S, S477N, T478K, E484A, Q493R, G496S, Q498R, N501Y, Y505H, T547K, D614G, H655Y, N679K, P681H, N764K, D796Y, N856K, Q954H, N969K, L981F
Omicron BA.2	T19I, △24/26, G142D, V213G, G339D,S371F, S373P, S375F, T376A, D405N, R408S, K417N, N440K, S477N, T478K, E484A, Q493R, Q498R, N501Y, Y505H, D614G, H655Y, N679K, P681H, N764K, D796Y, Q954H, N969K

Supplementary Figures

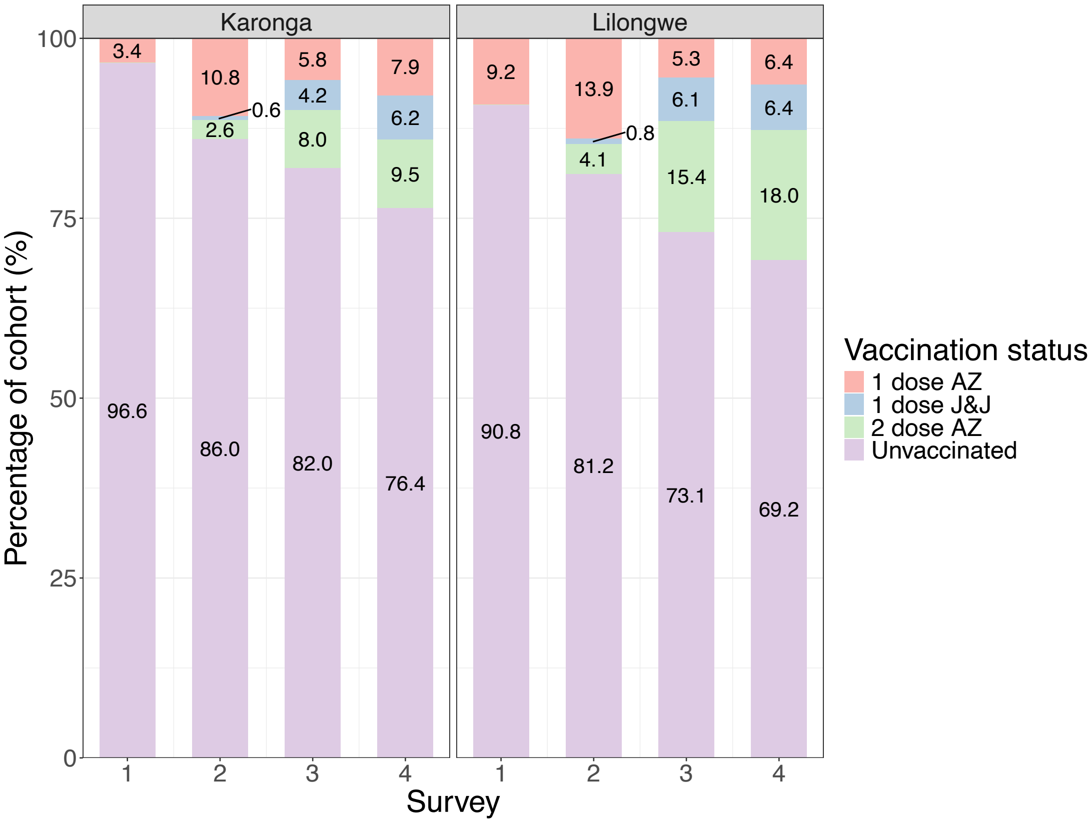

Supplementary Figure 1. COVID-19 vaccination coverage in Karonga and Lilongwe across the four study surveys (n=1,876). Percentage (%) of those vaccinated and unvaccinated, separated by vaccine type and number of doses received in Karonga (rural) and Lilongwe (urban). 1 dose AstraZeneca vaccine (AZ) – coral, 1 dose Johnson & Johnson (J&J) vaccine – blue, 2 doses AZ – green, Unvaccinated – purple. Sample sizes as follows: Karonga – Survey 1, n=774; Survey 2. n=731; Survey 3, n=710; Survey 4, n=673; Lilongwe – Survey 1, n=741; Survey 2, n=591; Survey 3, n=505; Survey 3, n=450.

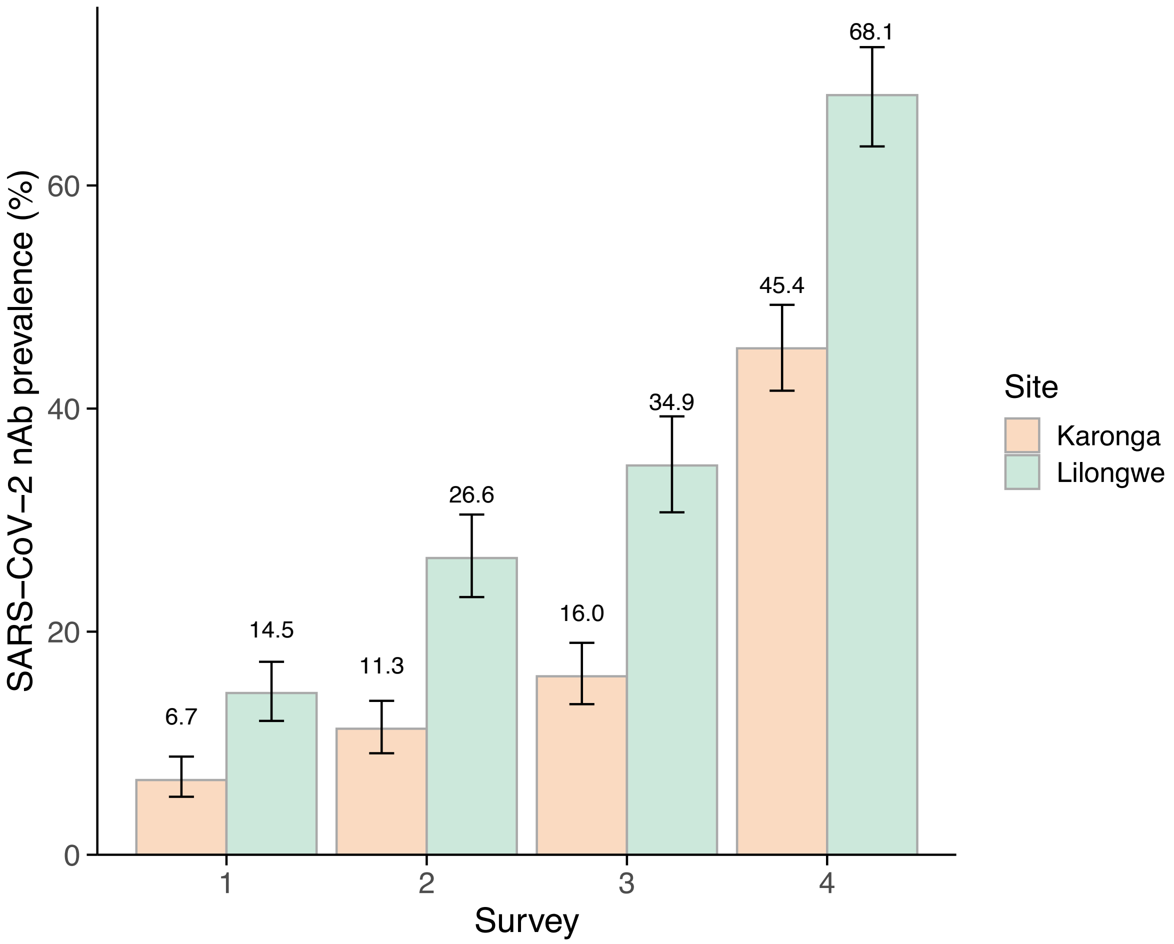

Supplementary Figure 2. SARS-CoV-2 neutralising antibody (nAb) prevalence in individuals from Karonga (orange) and Lilongwe (green) across the four study surveys. Percentage (%) of those who tested positive for nAb by single dilution screen using HIV(SARS-CoV-2) PVNA. Error bars are 95% confidence intervals (CI) for the seroprevalence. Restricted to HIV-uninfected participants: Karonga – Survey 1, n=741; Survey 2. n=700; Survey 3, n=680; Survey 4, n=643; Lilongwe – Survey 1, n=691; Survey 2, n=545; Survey 3, n=467; Survey 3, n=414.

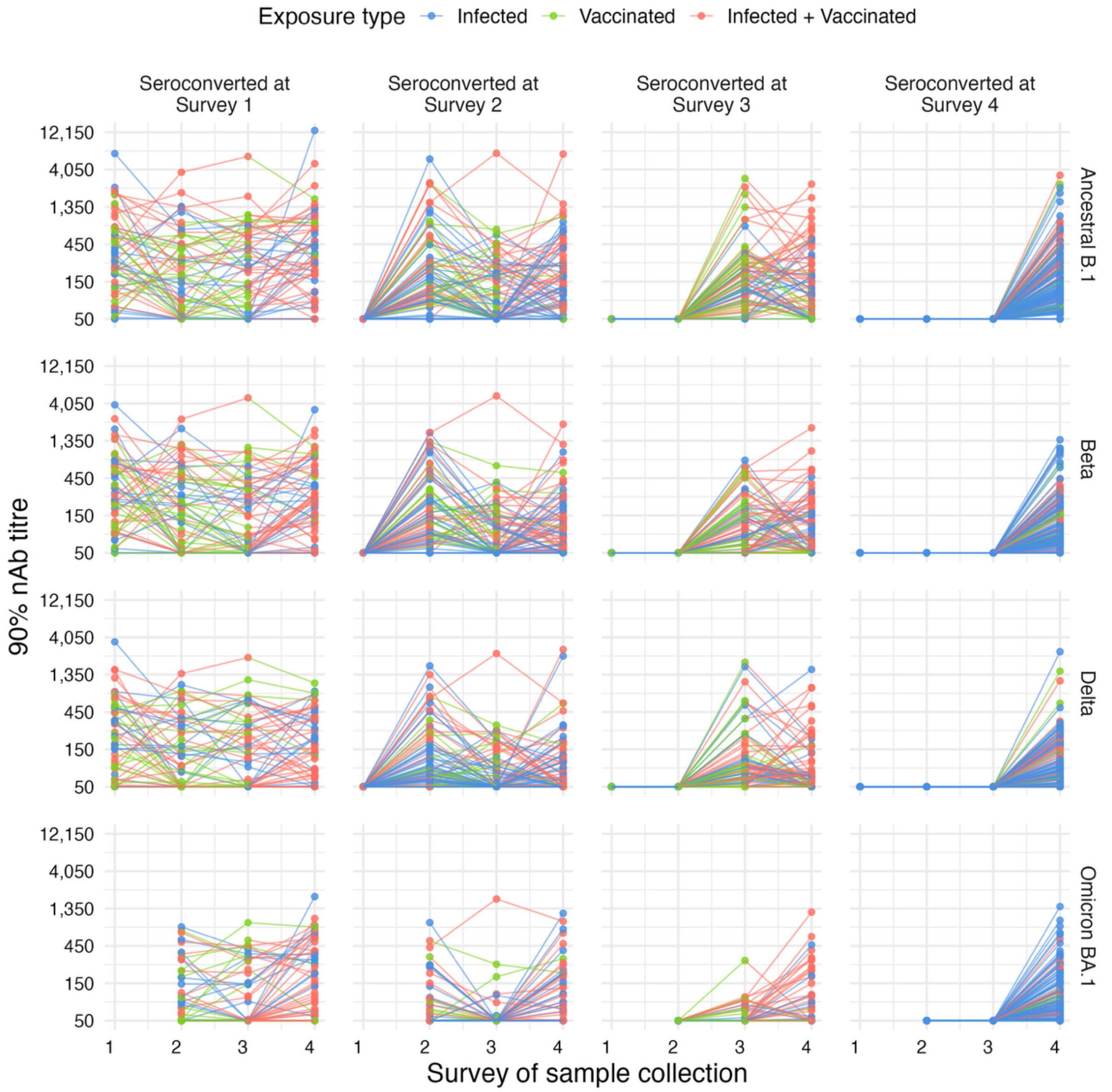

Supplementary Figure 3. Longitudinal neutralising antibody (nAb) trajectories per participant. Participants included are those who contributed a complete sample series (n=397) that were nAb seropositive at least once within the study time frame and were self-reported HIV-uninfected. The x-axis displays the survey of sample collection, and the y-axis displays the 90% nAb titre as measured using the HIV(SARS-CoV-2) PVNA. Participants are stratified by the survey at which they seroconverted (columns) and the SARS-CoV-2 virus they were tested against (rows). Only the results for Ancestral B.1, Beta, Delta, and Omicron BA.1 are displayed as Alpha and Omicron BA.2 were only tested against at Survey 1 and 4, respectively, Testing for Omicron BA.2 only began at Survey 2. Each dot displays the nAb titre for each participant, coloured by their exposure type at that type point (infected in blue, vaccinated in green, and infected + vaccinated in red). The lines connect samples from the sample participant at each study survey, coloured by exposure type at the point they are leading to.

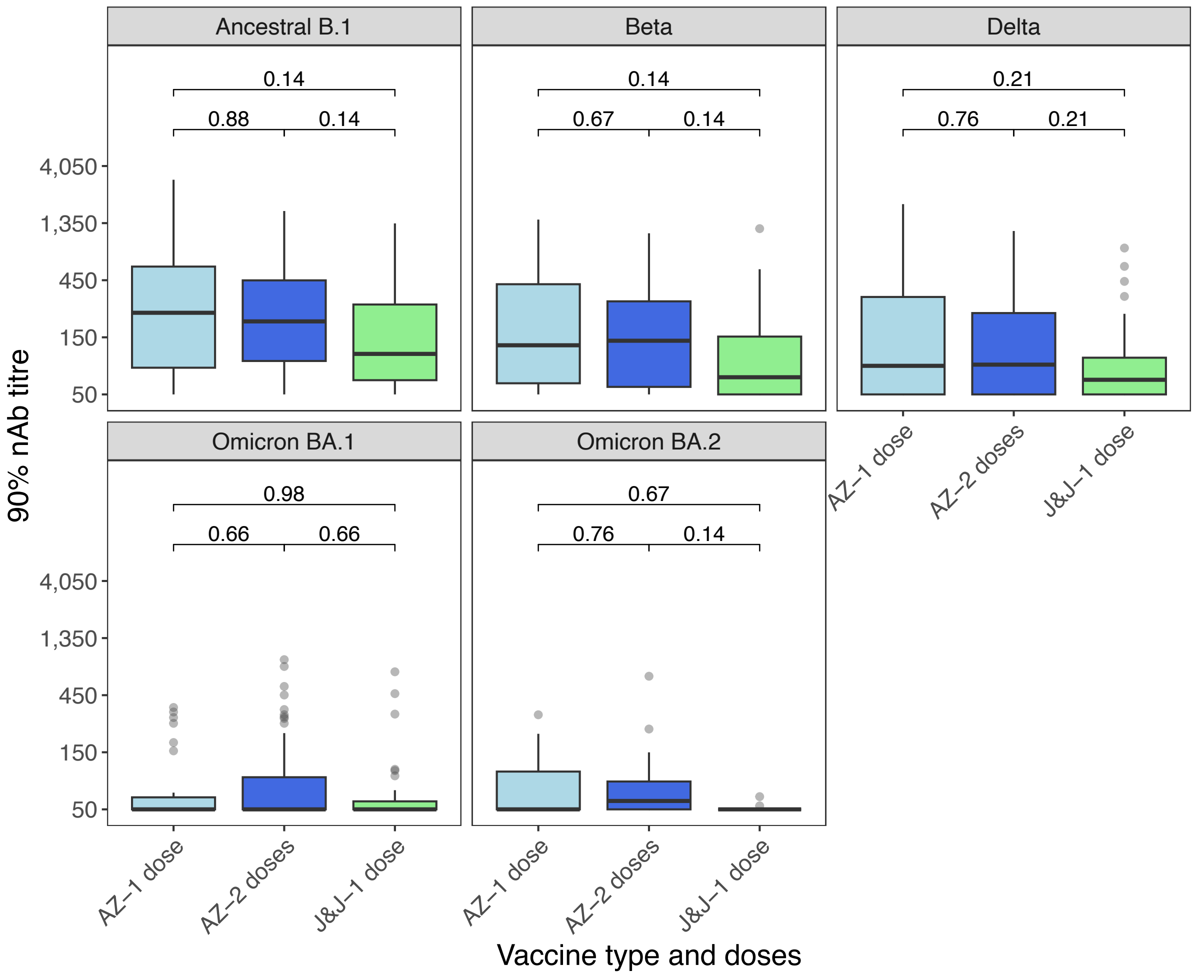

Supplementary Figure 4. Neutralising antibody titres by COVID-19 vaccination type. Boxplots comparing neutralising antibody (nAb) titres in those vaccinated with different vaccination types – AstraZeneca (AZ)-1 dose (light blue, n=50), AZ-2 doses (dark blue, n=72), Johnson & Johnson (J&J)-1 dose (green, n=12) - separated by the responses to the different variants. Samples included are from vaccinated participants who were positive for neutralising antibodies, study surveys combined (total n=134). Measurements were taken using HIV(SARS-CoV-2) PVNA. Boxplots display the median and interquartile range (IQR) of the outcome (90% titre). Statistical test used was Wilcoxon rank sum test (BH adjustment), with the p-value for the relationship between different groups displayed.

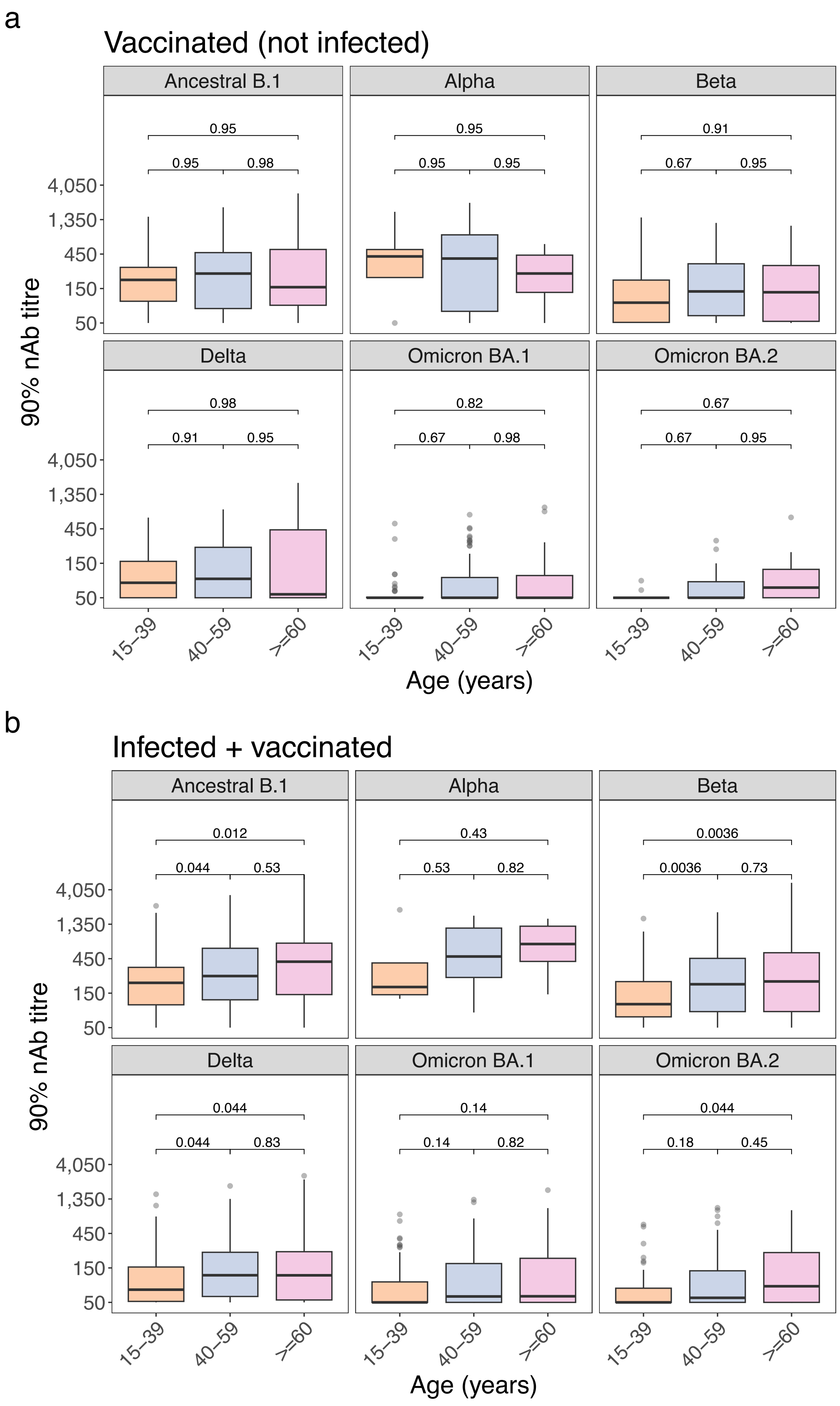
a
Vaccinated (not infected)
Ancestral B.1
Alpha
Beta
Delta
Omicron BA.1
Omicron BA.2
0.95
0.95
0.98
0.95
0.95
0.95
0.91
0.67
0.95
0.98
0.91
0.95
0.82
0.67
0.98
0.67
0.67
0.95
90% nAb titre
4,050
1,350
450
150
50
15–39
40–59
>=60
Age (years)
b
Infected + vaccinated
Ancestral B.1
Alpha
Beta
Delta
Omicron BA.1
Omicron BA.2
0.012
0.044
0.53
0.43
0.53
0.82
0.0036
0.0036
0.73
0.044
0.044
0.83
0.14
0.14
0.82
0.044
0.18
0.45
90% nAb titre
4,050
1,350
450
150
50
15–39
40–59
>=60
Age (years)

Supplementary Figure 5. Association between SARS-CoV-2 neutralising antibody (nAb) titre and age at sample collection. (A) nAb titres by age in individuals COVID-19 vaccinated (not SARS-CoV-2 infected), stratified by SARS-CoV-2 variant (n=134 - SARS-CoV-2 nAb positive samples from participants across the study surveys). Sample size for age groups as follows: 15-39, n=42 (orange); 40-59, n=67 (blue); >=60, n=25 (pink). (B) nAb titres by age in individuals SARS-CoV-2 infected and COVID-19 vaccinated (hybrid immunity), stratified by SARS-CoV-2 variant (n=210 - SARS-CoV-2 nAb positive samples from participants across the study surveys). Sample size for age groups as follows: 15-39, n=74 (orange); 40-59, n=89 (blue); >=60, n=47 (pink). Titres were measured using the HIV(SARS-CoV-2) PVNA. Those aged <15 years were excluded as they had not received COVID-19 vaccines. Box plots display the median and interquartile range (IQR) of the outcome (90% titre). Statistical test used was Wilcoxon rank sum test (BH adjustment), with the p-value for the relationship between different groups displayed.

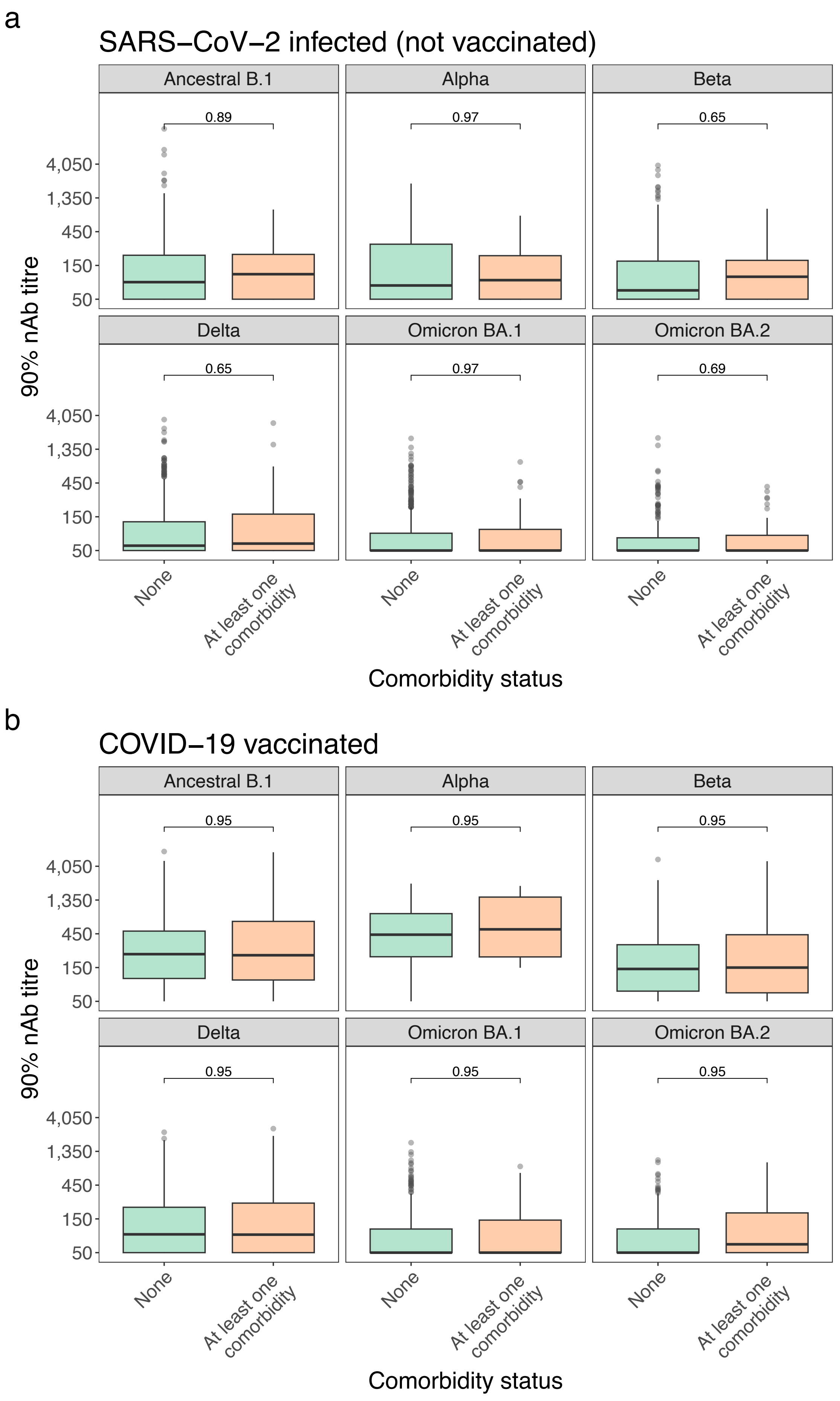
a
SARS−CoV−2 infected (not vaccinated)
Ancestral B.1
Alpha
Beta
Delta
Omicron BA.1
Omicron BA.2
0.89
0.97
0.65
0.65
0.97
0.69
4,050
1,350
450
150
50
90% nAb titre
None
At least one comorbidity
Comorbidity status
b
COVID−19 vaccinated
Ancestral B.1
Alpha
Beta
Delta
Omicron BA.1
Omicron BA.2
0.95
4,050
1,350
450
150
50
90% nAb titre
None
At least one comorbidity
Comorbidity status

Supplementary Figure 6: SARS-CoV-2 neutralising antibody (nAb) titre by comorbidity status. (A) nAb titres by comorbidity status in SARS-CoV-2 infected (not vaccinated) participants, stratified by SARS-CoV-2 variant. N=452 samples from individuals with no comorbidities (green), n=48 samples from individuals with comorbidities (orange) – study surveys combined. (B) nAb titres by comorbidity in individuals COVID-19 vaccinated (including those both SARS-CoV-2 infected and vaccinated (hybrid immune) and those solely vaccinated), stratified by SARS-CoV-2 variant. N=210 samples from individuals with no comorbidities (green), n=47 samples from individuals with comorbidities (orange) – study surveys combined. Titres were measured using the HIV(SARS-CoV-2) PVNA. Box plots display the median and interquartile range (IQR) of the outcome (90% titre). Statistical test used was Wilcoxon rank sum test (BH adjustment), with the p-value for the relationship between different groups displayed.

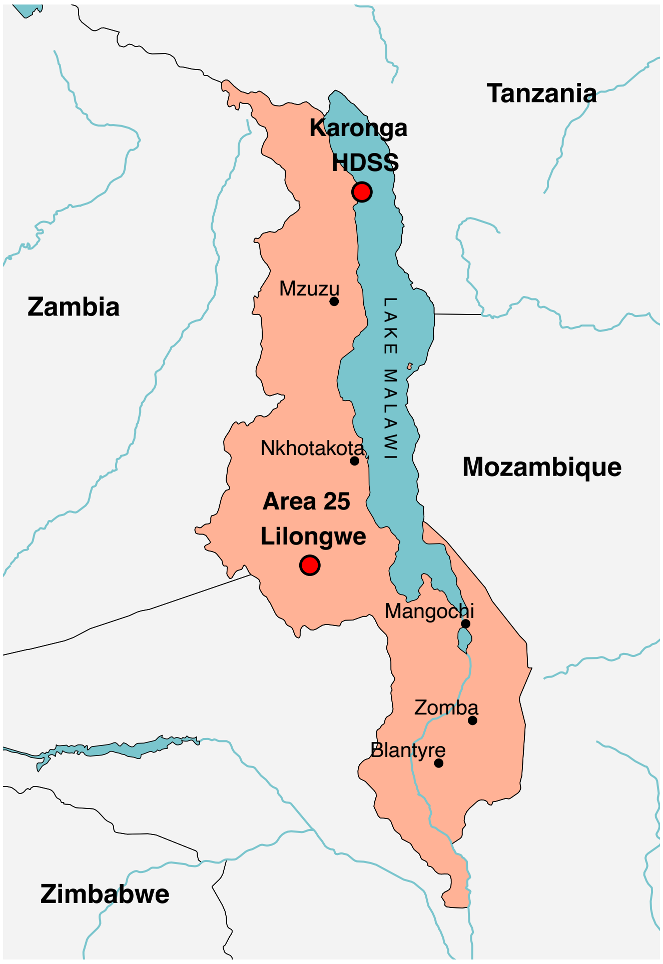

Supplementary Figure 7. Map of Malawi, showing the location of the Karonga Health Demographic Surveillance Site (HDSS) and Area 25, Lilongwe. Created with RStudio (rnaturalearth[3] and ggplot2[4] packages).

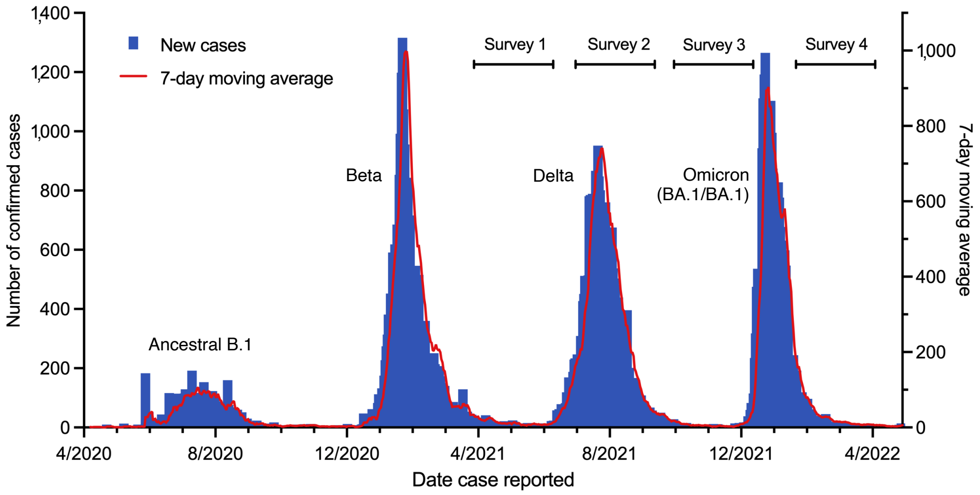

Supplementary Figure 8. Survey periods for the study. Number of laboratory-confirmed new cases of SARS-CoV-2 during each of the four survey periods (blue) in comparison with the 7 day moving average (red), data from the Public Health Institute of Malawi. The first, smaller peak is thought to be from the ancestral B.1 virus. Subsequent peaks are due to Beta, Delta and Omicron (BA.1/BA.2) variants. The amplitude of the ancestral B.1 peak versus the subsequent peaks should not be compared due to a lack of routine diagnostic testing at the time of the first wave in Malawi. Adapted from Banda et al. (2023)[5].

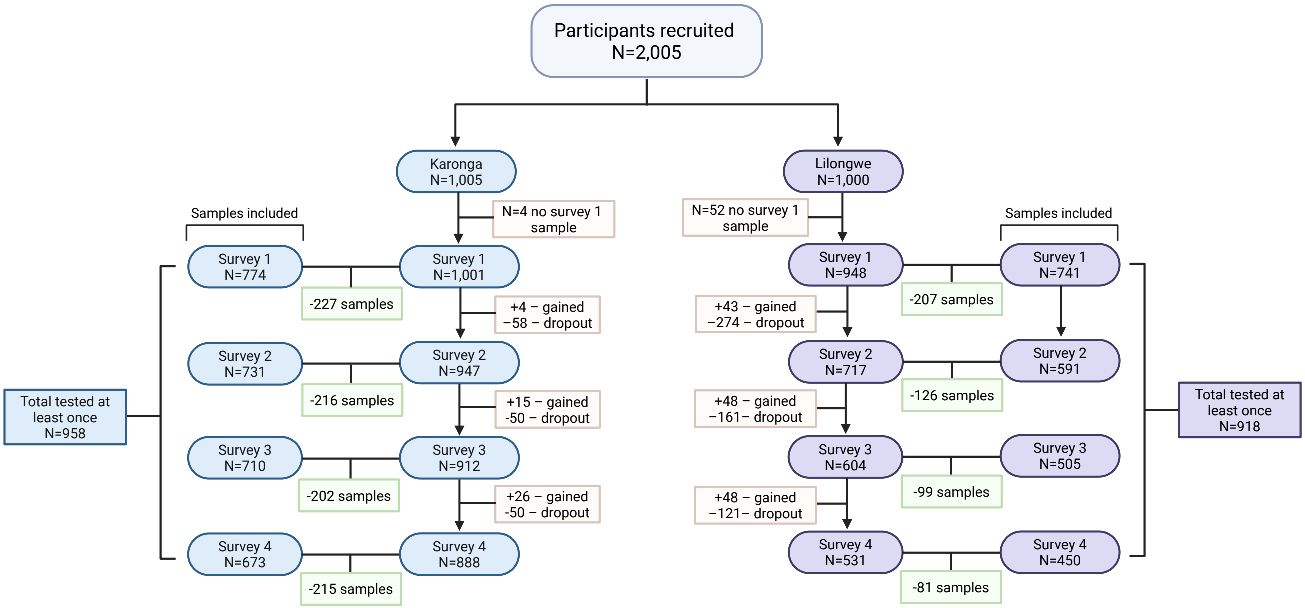

Supplementary Figure 9. Flow chart of study participants. Number of participants at each study survey and those included in this analyses, for Karonga (blue) and Lilongwe (purple). Survey 1: 24th February-8th June 2021; Survey 2: 28th June-13th September 2021; Survey 3: 4th October-10th December 2021; Survey 4: 27th January-22nd April 2022. The number of participants gained and lost (due to dropout) between successive surveys is detailed (orange). Green boxes indicate the number of participants not included in these analyses per survey. Reasons for exclusion include: no sample received at Glasgow based laboratory (commonly due to limited blood volume collected from children); insufficient serum volume for testing; labelling issues that prevented linking of the sample to participant metadata. The number tested of participants tested at least once exceeds the number in any individual survey, as individuals entered and exited the study over time.

Supplementary References

1. Zufferey, R. et al. Multiply attenuated lentiviral vector achieves efficient gene delivery in vivo. *Nat. Biotechnol.* **15**, 871-875; 10.1038/nbt0997-871(1997).
2. Zufferey, R. et al. Self-Inactivating Lentivirus Vector for Safe and Efficient In Vivo Gene Delivery. *J. Virol.* **72**, 9873-9880; 10.1128/JVI.72.12.9873-9880.1998 (1998).
3. Massicotte, P. et al. rnaturalearth: World Map Data from Natural Earth (version 1.0.1). rOpenSci (2023).
4. Wickham, H. ggplot2: Elegant Graphics for Data Analysis (version 3.5.1). Springer-Verlag, New York (2016).
5. Banda, L. et al. Characterizing the evolving SARS-CoV-2 seroprevalence in urban and rural Malawi between February 2021 and April 2022: A population-based cohort study. *Int. J. Infect. Dis.* **137**, 118–125; 10.1016/j.ijid.2023.10.020 (2023).
